# Development of rat and mouse models of heme-iron absorption

**DOI:** 10.1172/jci.insight.184742

**Published:** 2025-06-09

**Authors:** Jennifer K. Lee, Yue He, Shireen R.L. Flores, Regina R. Woloshun, Xiaoyu Wang, Jacob S. Shine, Pearl O. Ebea-Ugwuanyi, Sitara Sriram, Melissa Fraga, Sean Zhu, Yang Yu, Iqbal Hamza, James F. Collins

**Affiliations:** 1 Food Science & Human Nutrition Department, University of Florida, Gainesville, Florida, USA.; 2 Center for Blood Oxygen Transport & Hemostasis, School of Medicine, University of Maryland, Baltimore, Baltimore, Maryland, USA.; 3 Department of Animal and Avian Sciences, University of Maryland, College Park, Maryland, USA.

**Keywords:** Gastroenterology, Hematology, Metabolism, Homeostasis, Mouse models

## Abstract

Heme iron (HI), derived principally from hemoglobin (Hb) in animal foods, is a highly bioavailable source of dietary iron for humans. Despite several decades of focused research, however, molecular mechanisms governing HI absorption remain undefined. Previous studies in mice and rats have not produced a consensus, definitive model of efficient HI absorption/utilization. We hypothesized that a nutritional approach, using semipurified, HI-containing diets, could be utilized to establish a tractable rodent model of HI absorption that could ultimately be employed to test the roles of receptors, transporters, and enzymes using genetic engineering technology. Experiments were designed to assess HI utilization by feeding animals AIN-93G–based, HI-enriched experimental diets formulated with lyophilized porcine RBCs, containing approximately 85% HI and 15% nonheme iron (NHI). Total iron was within the physiological range (50–75 ppm) and precisely matched NHI control diets containing ferrous sulfate were utilized as comparators. Notably, in Sprague-Dawley (S-D) rats and C57BL/6 (B6) mice, dietary HI effectively (a) resolved iron-deficiency anemia; (b) supported normal pregnancy, lactation, and neonatal development; and (c) contributed to iron loading in *Hamp*-KO mice and rats (modeling hereditary hemochromatosis). A nutritional paradigm has thus been established that facilitates investigation into mechanisms of HI absorption by S-D rats and B6 mice.

## Abstract 2

A nutritional paradigm has thus been established that facilitates investigation into mechanisms of heme iron absorption by Sprague-Dawley rats and C57BL/6 mice.

## Graphical abstract

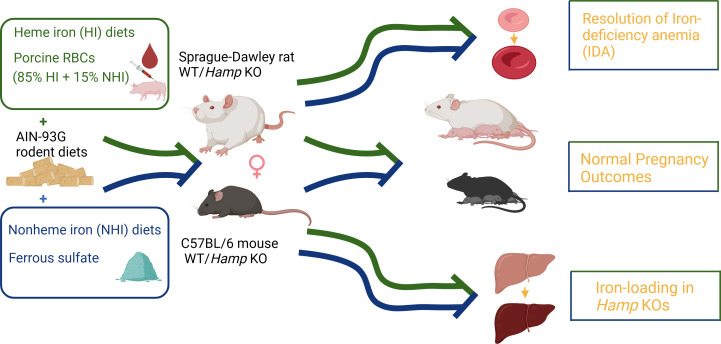

## Introduction

Iron in the human diet is mainly nonheme iron (NHI), present in foods of plant and animal origin, and heme iron (HI), derived principally from hemoglobin (Hb) and myoglobin in animal products. Pathways of NHI absorption have been recently clarified (1–3), but mechanisms governing HI absorption remain uncertain (4, 5). Our current, albeit incomplete, understanding of dietary HI assimilation posits the following: (a) Heme is released from dietary Hb, myoglobin, and hemoproteins in the GI tract by the concerted actions of gastric acid and luminal proteases. (b) Free heme binds to a receptor, or transporter, on the brush-border membrane of enterocytes of the small bowel as an intact metalloporphyrin [Fe(II)-protoporphyrin-IX] (6). (c) Heme is internalized by endocytosis and then transported out of endosomes into the cytosol by a specific carrier protein (6). A possible transporter purported to fulfill this function is heme carrier protein 1 (HCP1) (7); however, HCP1 is now generally recognized as a high-affinity folate transporter (8). (d) Cytosolic heme is catabolized by heme oxygenase 1 (HMOX1) in the endoplasmic reticulum (9), thus liberating iron from the protoporphyrin ring, which then joins the cytosolic labile iron pool along with iron derived from dietary NHI absorption. (e) Iron derived from HI and NHI is exported from enterocytes by the basolateral membrane (BLM) iron transporter ferroportin (FPN) (10, 11). A common export mechanism for both forms of dietary iron suggests that the overall process is coordinately regulated by hepcidin, which controls iron efflux from enterocytes by modulating FPN activity on the BLM.

Alternative HI absorption pathways have also been proposed, including the possibility that heme is catabolized within the endosomal pathway (12, 13). In this case, after endocytosis, heme would be catabolized within endosomes by HMOX1/2 or an unidentified protein, and then the ferrous iron liberated from the protoporphyrin ring is transported out of endosomes/lysosomes into the cytosol by an NHI transporter, such as DMT1 (14). Another possibility is that heme is exported from enterocytes intact (12, 13), possibly by FLVCR, ABCG2, or unknown heme transporters (15, 16), followed by transfer to a specific carrier protein such as hemopexin (17) in the portal circulation, and then catabolized by HMOX in the liver (13).

Seminal studies on HI absorption and utilization, first being reported in the 1950s and 1960s, were carried out in dogs (18, 19) and pigs (20, 21), and several human studies have been completed as well (12, 22–25). The use of large animal models may not be feasible for many researchers, however, and experimentation in humans comes with certain challenges. In the past 3 decades, most research on iron biology has utilized mice (26, 27). However, since mice are generally thought to poorly absorb and utilize dietary iron derived from heme (28, 29), development of alternative models is warranted. Recently published studies demonstrated that rats could absorb dietary HI, but with variable efficiency being reported across studies (29–38). This variability could be due to the experimental approaches utilized or to intrinsic, interstrain differences. A critical analysis of previous experimental work in this area led us to hypothesize that using a physiological/nutritional approach could accelerate development of a suitable rat model of HI absorption. Accordingly, HI-enriched, AIN-93G–based (semipurified) diets were formulated with lyophilized porcine red blood cells (RBCs) and fed to experimental animals ad libitum. Total iron content of the diets was within the adequate range for rodents, and precisely matched NHI diets containing ferrous sulfate were utilized as internal controls. Moreover, given the similar natural history of rats and mice, being opportunistic omnivores, we postulated that the same nutritional approach could lead to development of a suitable mouse model of HI absorption. Performing parallel studies in mice was important for 2 reasons: (a) the concept that mice are poor absorbers of dietary HI is supported by a paucity of rigorous experimentation; and (b) genetically engineered mouse models are readily available, which could accelerate discovery. Development of rat and mouse models of HI absorption involved 3 distinct phases, which tested the ability of dietary HI to (a) correct iron-deficiency anemia (IDA) in WT rats and mice; (b) provide the extra iron required to support pregnancy, lactation, and neonatal development in WT animals; and (c) contribute to iron loading in hepcidin-KO (*Hamp*-KO) rats and mice, modeling hereditary hemochromatosis (HH). Collectively, these experiments revealed efficient absorption and utilization of dietary HI by Sprague-Dawley (S-D) rats and C57BL/6 (B6) mice.

## Results

### Iron depletion-repletion studies in rats.

**Figure 1:**
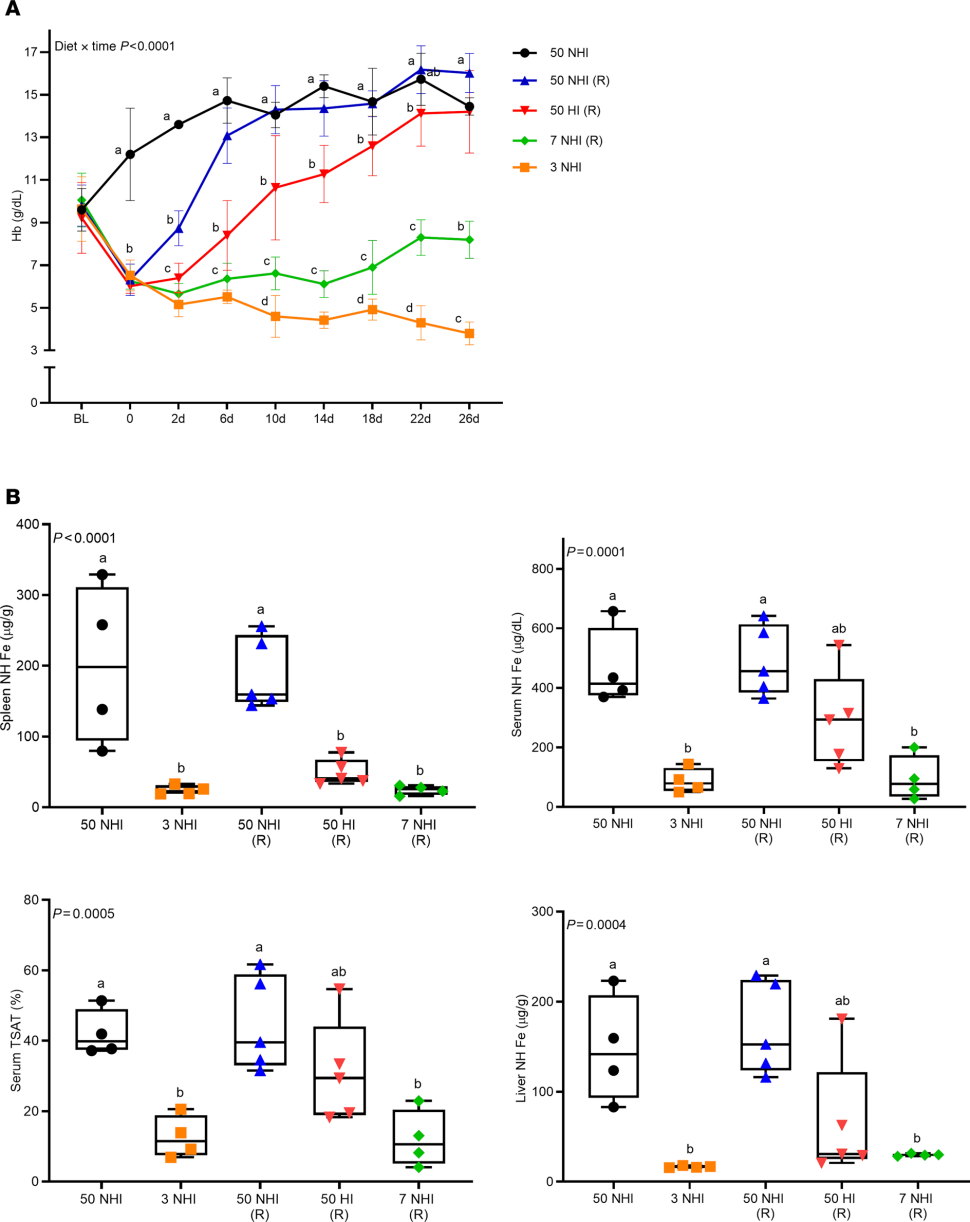
Rat pilot study 2: An HI diet was effective at correcting the anemia in iron-depleted, S-D rats. Weanling, female rats were fed a low-iron diet for 2 weeks (~3 ppm NHI) and then repleted (R) with a 7-ppm NHI (internal control) diet, a 50-ppm NHI (control) diet, or a 50-ppm HI–enriched (experimental) diet. Additional control groups were fed the iron-adequate (50 ppm NHI) diet or the low-iron diet (3 ppm NHI) throughout. Rats were bled every 4 days and sacrificed 26 days after initiation of the iron-repletion phase. Shown are Hb levels at each time point (**A**) and tissue/serum NHI levels and TSAT at the end of the study (**B**). Data are mean ± SD for *n* = 4–5 rats per group and were analyzed by 2-way (**A**) or 1-way (**B**) ANOVA followed by Tukey’s multiple-comparison test. Groups with different letters vary significantly. Significant 2-way interaction (**A**) and main effect (**B**) *P* values are indicated in each panel. **A**: BL, baseline, prior to iron deprivation; 0, after 2 weeks of low-iron feeding; 2 days, 6 days, etc., indicates repletion period in days.

In rat pilot study 1, the ability of dietary HI to mitigate IDA was assessed in 3 common strains of laboratory rats (*Rattus norvegicus*): Wistar, Lewis, and S-D. Wistar and S-D rats are outbred, while Lewis rats are inbred. Results revealed that the S-D rat exhibited the most significant increase in blood Hb levels upon short-term repletion with an HI-enriched diet (Supplemental Figure 1; supplemental material available online with this article; https://doi.org/10.1172/jci.insight.184742DS1), so this strain was selected for further experimentation. In rat pilot study 2, a longer-term experiment was undertaken. Results demonstrated that repletion of iron-deficient, anemic S-D rats with a 50-ppm NHI diet or an HI-enriched diet with 50 ppm total iron both effectively resolved IDA. Although the Hb recovery rate was apparently slower in rats consuming the HI diet, Hb levels were normalized in both dietary groups by the end of the 26-day repletion period (Figure 1A). Both diets also restored serum (transport) iron and liver (storage) iron and transferrin saturation (TSAT) to control levels. Spleen iron, however, was not fully replenished in the HI diet–fed rats (Figure 1B), suggesting that the animals remained moderately iron deficient. These experiments provided the rationale for further testing and development of the S-D rat as a model of HI absorption/utilization.

**Figure 2:**
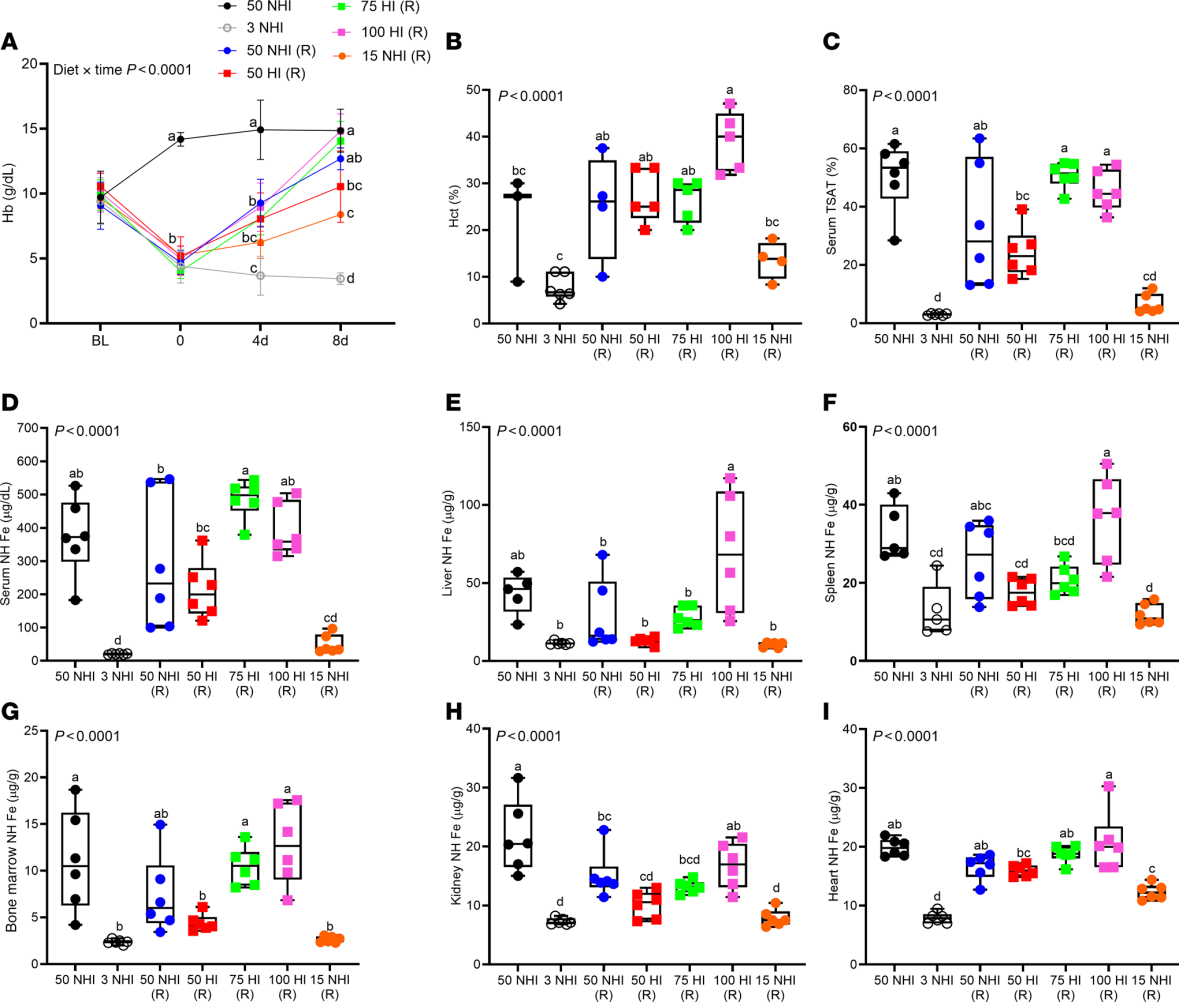
The 50-ppm NHI and 75-ppm HI repletion diets were equally effective at correcting IDA in S-D rats. Weanling, female rats were fed a low-iron diet (3 ppm NHI) for 2 weeks and then repleted (R) with 50-, 75-, or 100-ppm HI–enriched experimental diets, or a 50-ppm NHI (iron adequate) control diet. The 15-ppm NHI (internal control) diet matched the NHI content of the 100-ppm HI–enriched diet (i.e., 85 ppm HI and 15 ppm NHI). Additional control groups were fed the iron-adequate or the low-iron diet throughout. Rats were bled every 4 days and sacrificed 8 days after initiation of the iron-repletion phase. Hb levels are shown at each time point (**A**). Also shown are Hct (**B**), serum TSAT (**C**), and serum (**D**), liver (**E**), spleen (**F**), bone marrow (**G**), kidney (**H**) and heart (**I**) NHI levels in experimental rats at the termination of the experiment. Results are presented as a line graph (**A**) or box-and-whisker plots (**B**–**I**) for *n* = 3–6 rats per group. Data were analyzed by 2-way (**A**) or 1-way (**B**–**I**) ANOVA followed by Tukey’s multiple-comparison test. Groups with different letters vary significantly. Significant main effect and 2-way interaction *P* values are shown in each panel. **A**: BL, baseline, prior to iron deprivation; 0, after 2 weeks of low-iron feeding; 4d and 8 d indicate repletion period in days.

**Figure 3:**
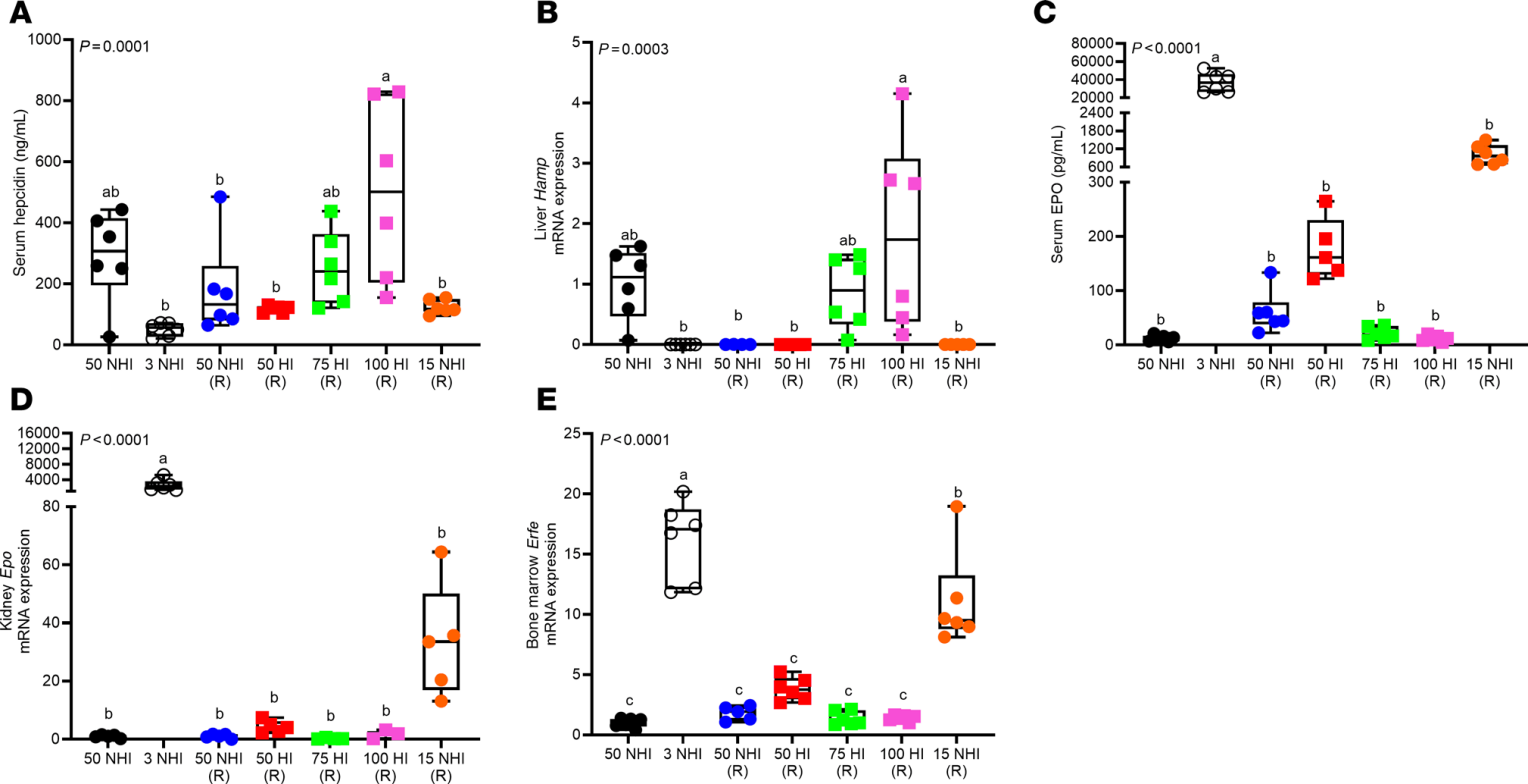
A 75-ppm HI diet was effective at normalizing serum hepcidin and EPO levels in iron-deprived S-D rats. Experimental details are the same as for those described in the Figure 2 legend. Shown are serum hepcidin (**A**), liver hepcidin mRNA expression (**B**), serum EPO (**C**), kidney Epo mRNA expression (**D**), and bone marrow *Erfe* mRNA expression (**E**). Results are presented as box-and-whisker plots for *n* = 3–6 rats per group. Data were analyzed by 1-way ANOVA followed by Tukey’s multiple-comparison test. Groups with different letters vary significantly. Significant main effect *P* values are shown in each panel. (R), repletion group.

Next, since the 50-ppm HI diet did not fully correct the iron deficiency in iron-depleted rats, a subsequent experiment utilized HI-enriched diets containing total iron up to 100 ppm, which is still within the upper range of physiological intakes but well below the iron content of most commercial rodent chows (which may contain ≥300 ppm Fe). Outcomes of this experiment showed that a 50-ppm NHI (control) repletion diet, and 75- and 100-ppm HI–enriched (experimental) repletion diets, all corrected the anemia within 8 days (Figure 2A); however, the 50-ppm HI and 15-ppm NHI repletion diets did not. The 50-ppm NHI repletion diet, and the 75- and 100-ppm HI repletion diets, were also equally effective at restoring hematocrit (Hct), TSAT, serum, liver, spleen, bone marrow, kidney, and heart iron levels (Figure 2, B–I), as well as serum hepcidin and liver hepcidin, renal erythropoietin (*Epo*), and bone marrow erythroferrone (*Erfe*) mRNA expression (Figure 3). ERFE is hormone that is secreted by developing erythrocytes in the bone marrow, under the influence of EPO, that downregulates *Hamp* gene transcription in hepatocytes, thus reducing circulating hepcidin levels (39).

Collectively, these experiments revealed that a 75-ppm HI–enriched diet, containing approximately 64 ppm HI and 11 ppm NHI, provided sufficient iron to fully mitigate severe IDA in adolescent S-D rats. Since the internal control (15 ppm) NHI diet was ineffective at correcting the IDA in these rats, we concluded that S-D rats efficiently assimilated and utilized iron derived from heme.

### Iron depletion-repletion studies in mice.

**Figure 4:**
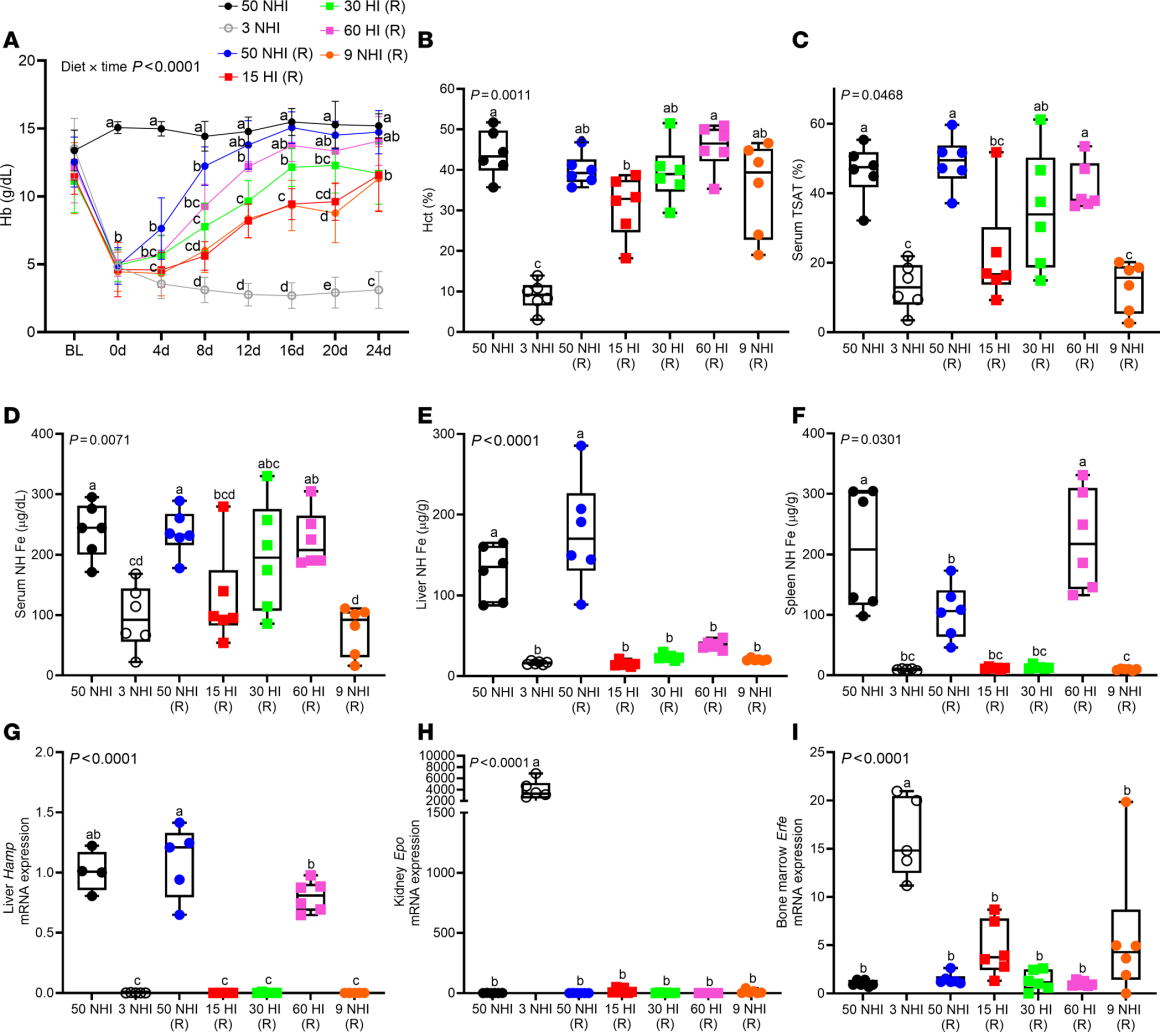
A 60-ppm HI diet was effective at correcting the IDA in iron-depleted C57BL/6 mice. Female weanling mice were fed a low-iron diet (3 ppm NHI) for 3 weeks and then switched to control repletion diets (50 ppm and 9 ppm NHI), or 1 of 3 different HI-enriched (experimental) repletion diets (15, 30, or 60 ppm iron). Additional control groups consumed an iron-adequate diet (50 ppm NHI) or the low-iron diet throughout. Mice were bled every 4 days and sacrificed 24 days after initiation of the iron-repletion phase. Hb levels are shown at each time point (**A**). Also shown are Hct (**B**), serum TSAT (**C**), and serum (**D**), liver (**E**) and spleen (**F**) NHI from mice at the termination of the experiment. Also shown are liver *Hamp* (**G**), kidney *Epo* (**H**), and bone marrow *Erfe* mRNA expression (**I**). Results are presented as a line graph (**A**) or box-and-whisker plots (**B**–**I**) for *n* = 5–6 mice per group. Data were analyzed by 2-way (**A**) or 1-way (**B**–**I**) ANOVA followed by Tukey’s multiple-comparison test. Groups with different letters vary significantly. Significant 2-way interaction (**A**) and main effect (**B**–**I**) *P* values are shown in each panel. **A**: BL, baseline, prior to iron deprivation; 0, after 3 weeks of low-iron feeding; 4d, 8d, etc., indicate repletion (R) period in days.

Encouraged by outcomes in S-D rats, we next assessed HI assimilation by iron-depleted B6 mice, a well-established mouse strain for iron metabolism studies. The rationale for studying mice was 2-fold: (a) HI absorption has been described as an inefficient process in mice (28), based on scant experimental evidence, and this is now the generalized perception in the field. As such, no extensively characterized mouse models of HI absorption have been developed to date; and (b) a multitude of genetically engineered mouse strains are commercially available, which could accelerate future discovery. We first sought to define the optimal dietary iron concentration to use for mouse experiments. In mouse pilot study 1, experimental HI-enriched diets containing 50–100 ppm total iron were utilized, since we postulated that dietary iron requirements would be similar for rats and mice. However, outcomes demonstrated that all of the experimental HI-enriched diets, and the control 50-ppm NHI diet, rapidly corrected IDA in anemic B6 mice (Hb ~11 g/dL) within 8 days (Supplemental Figure 2). This suggested that dietary iron should be lowered for experiments in mice, so the HI-enriched diets were reformulated with total iron ranging from 15 to 30 ppm. These diets were fed to iron-deficient, anemic mice (Hb ~5 g/dL) in mouse pilot study 2. Notably, this level of anemia better reflected the severe anemia in the rat iron depletion/repletion experiments described above. Results of this experiment demonstrated that 30 ppm HI was insufficient to fully restore Hb levels (Supplemental Figure 3), so diets were reformulated again with total iron up to 60 ppm. These dietary iron levels turned out to be appropriate, as the 60-ppm HI repletion diet increased blood Hb in iron-deprived, anemic mice (Hb ~5 g/dL) at essentially the same rate as the control 50-ppm NHI repletion diet (Figure 4A). The 15- and 30-ppm HI diets, and the 9-ppm NHI internal control diet, however, did not correct the anemia, even after 24 days of repletion. The 60-ppm HI–enriched diet also normalized Hct, TSAT, and serum and spleen iron levels, just as effectively as the iron-adequate, 50-ppm NHI diet (Figure 4, B–D, and F). None of the experimental HI diets (including the 100-ppm HI diet used in mouse pilot study 1), however, were able to fully replenish liver iron stores (Figure 4E). The 60-ppm HI diet also normalized liver *Hamp* and bone marrow *Erfe* mRNA expression (Figure 4, G and I). Renal *Epo* mRNA expression did not vary significantly between groups (Figure 4H). B6 mice thus effectively utilized dietary HI to recover from severe IDA.

### HI utilization during pregnancy by S-D rats and B6 mice.

Extra dietary iron is required to support pregnancy, lactation, and neonatal development in humans and rodents. To investigate whether dietary heme could provide this extra iron, we utilized an experimental 50-ppm HI–enriched diet, containing approximately 43 ppm HI and 7 ppm NHI, and an iron-adequate, 50-ppm NHI control diet. Importantly, the minimal amount of NHI iron recommended for rats and mice in the AIN-93G diet is 35 ppm (40, 41), which is formulated for the growth, pregnancy, and lactation phases of development. The 7-ppm background NHI in the 50-ppm HI–enriched diet is thus well below this minimum requirement, so inclusion of a 7-ppm NHI internal control diet was unnecessary. Female S-D rats were fed these diets for 1 week prior to mating and conception, and throughout pregnancy and lactation. Outcomes showed that many parameters were invariable between dietary groups, including the number and sex distribution of pups, blood Hb levels and TSAT in dams and pups, as well as serum, liver, and spleen NHI in pups (Tables 1 and 2). Some differences were also noted between groups, including lower body weights in dams (~20%) and pups (20%–25%) in the HI groups. Dams consuming the HI diet also had lower serum iron (by ~40%), but surprisingly, liver (by ~3-fold) and spleen (by ~2-fold) iron were elevated significantly, as compared with dams fed the NHI diet. Tissue distribution of iron thus varied by dietary source (HI vs. NHI), consistent with findings from a recent publication (31). Increasing total iron up to 75 ppm may have prevented the noted growth impairment in dams and pups, since this amount of dietary iron was the minimal amount requirement to fully support recovery from severe IDA in S-D rats (Figures 3 and 4).

An identical pregnancy study in B6 mice showed that there were no noted differences in any measured parameters in dams consuming either diet (Table 3). In the pups, almost all outcomes were also invariable between dietary groups, with the only difference being a 13% (possibly unimportant) reduction in Hb levels in males (Table 4). The 50-ppm HI diet thus fully supported normal fertility, pregnancy, lactation, and development of neonates in mice. Collectively, these data demonstrated that S-D rats and B6 mice efficiently utilized iron derived from dietary Hb/heme to meet the enhanced iron requirements necessary to support successful pregnancy outcomes.

### Defining iron requirements for Hamp-KO B6 mice and S-D rats, and iron loading study.

HH is a heritable, iron-loading disorder in humans, most commonly caused by mutations in the *HFE* gene (42). Mutations in *HAMP*, encoding hepcidin, underlie a rare, severe early-onset form of the disease (43, 44). In HH, iron overload ensues due to low hepcidin, which results in inappropriately elevated intestinal iron absorption and pathological iron accumulation in parenchymal tissues. Evidence in the scientific literature supports the concept that dietary HI and NHI are both hyperabsorbed in patients with HH (45, 46). *Hamp*-KO mice are an excellent model of HH (47). Since our studies revealed efficient HI absorption and utilization by rats and mice, we next examined whether dietary HI could also precipitate iron loading in *Hamp*-KO S-D rats (48) and B6 mice. However, it was first necessary to define the dietary iron requirements for *Hamp*-KO rats and mice, since they hyperabsorb dietary iron.

**Figure 5:**
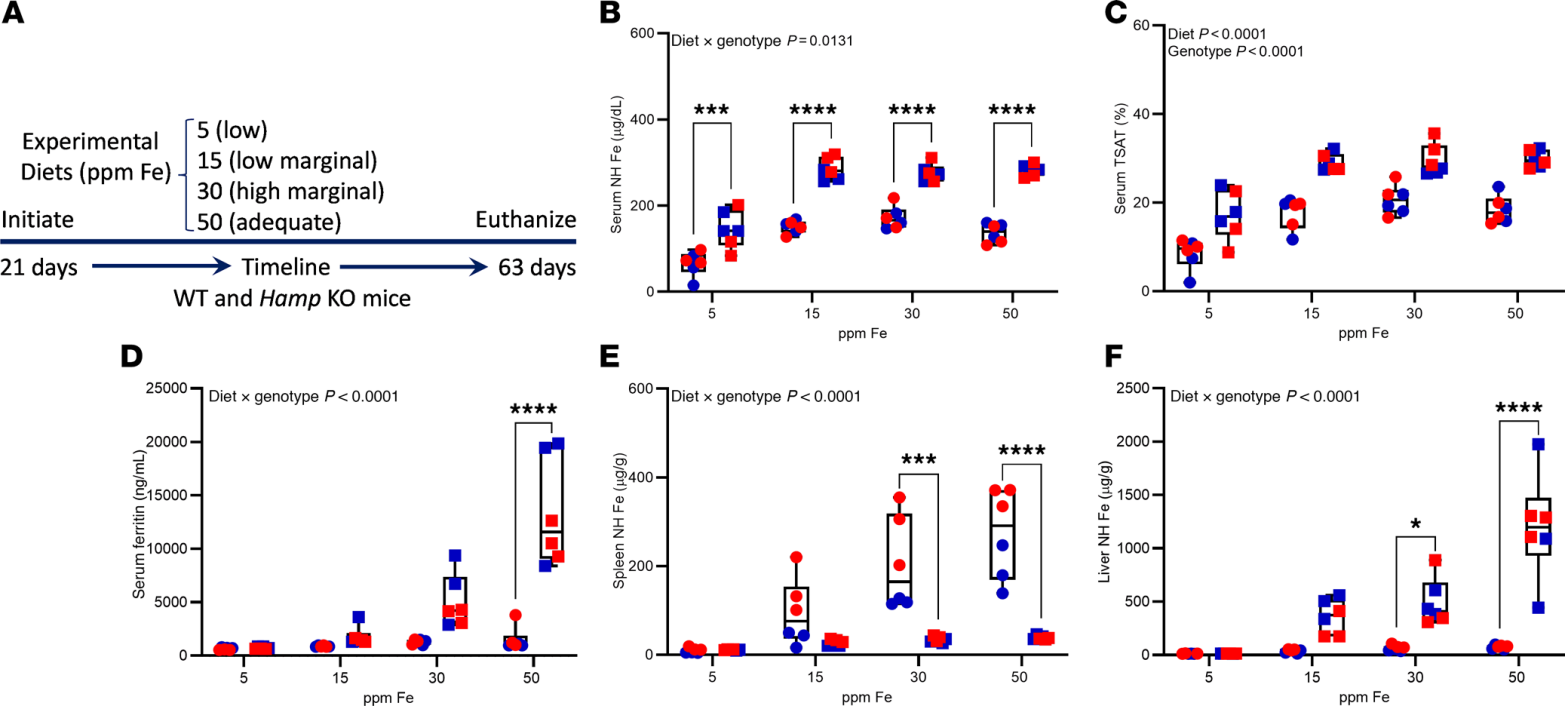
Consumption of a 15-ppm NHI diet prevents iron overload in *Hamp*-KO C57BL/6 mice. Male (blue symbols) and female (red symbols) WT (circles) and *Hamp*-KO (squares) mice were fed diets with variable NHI content for 6 weeks after weaning. Shown are experimental design (**A**), serum NHI levels (**B**), serum TSAT (**C**), serum ferritin (**D**), and spleen (**E**) and liver (**F**) NHI content. Data are presented as box-and-whisker plots for *n* = 6 mice/group and were analyzed by 2-way ANOVA followed by Tukey’s multiple-comparison test. Asterisks denote significant differences between the WT and KOs within a diet group. *P < 0.05; ***P < 0.001; ****P < 0.0001. Significant 2-way interaction and main effect *P* values are shown in each panel.

**Figure 6:**
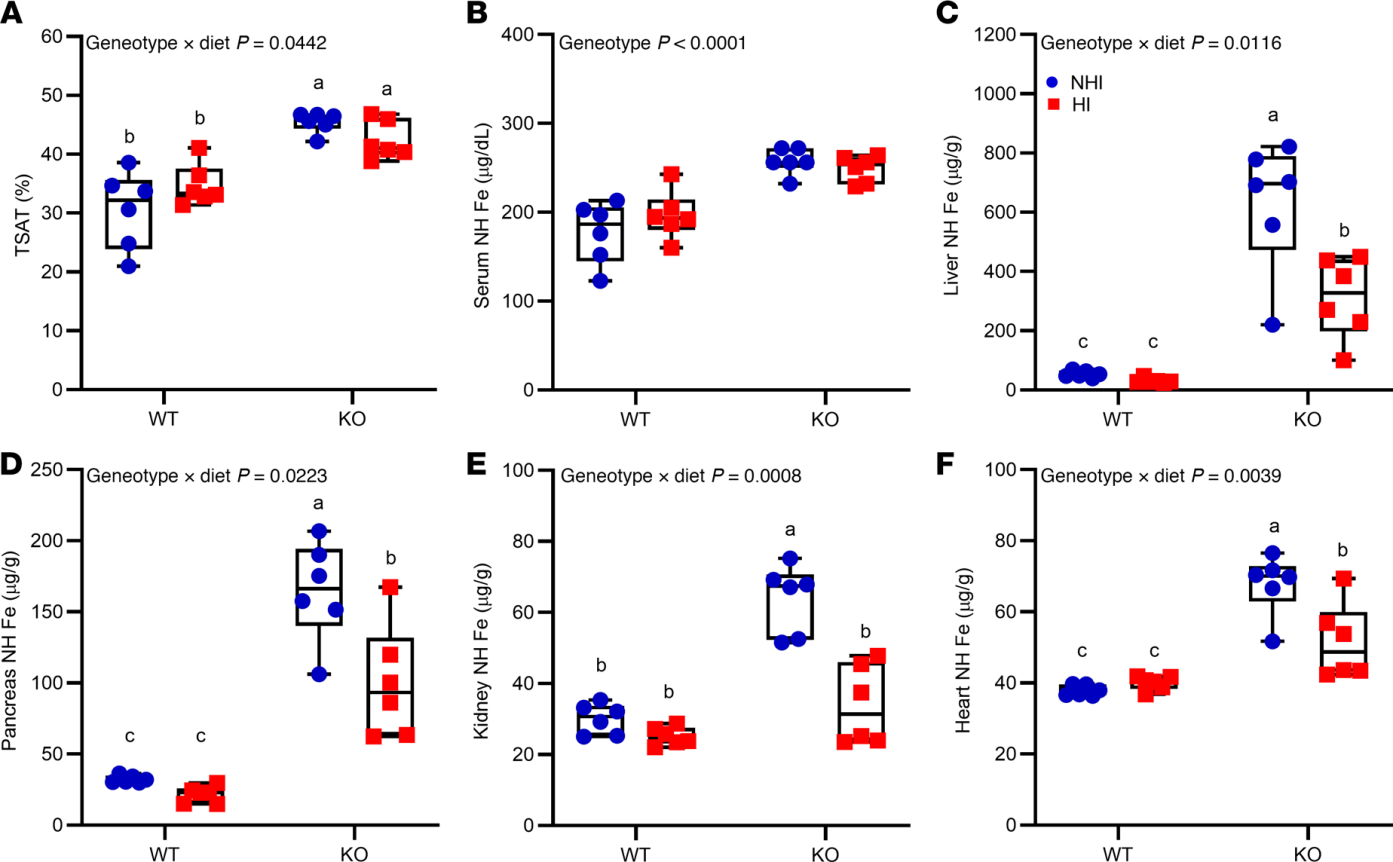
Consumption of a 50-ppm NHI diet or a 50-ppm HI–enriched diet leads to iron loading in *Hamp*-KO C57BL/6 mice. Female WT and *Hamp*-KO mice were weaned onto a control diet with 50 ppm NHI (blue circles), or an experimental 50-ppm HI–enriched diet (red squares), and then sacrificed 6 weeks later. Shown are serum TSAT (**A**), and serum (**B**), liver (**C**), pancreas (**D**), kidney (**E**), and heart (**F**) NHI levels, which were determined in experimental mice at the termination of the experiment. Data were analyzed by 2-way ANOVA followed by Tukey’s multiple-comparison test. Results are presented as box-and-whisker plots for *n* = 6 mice per group. Groups with different letters vary significantly. Significant 2-way interaction and main effect *P* values are shown in each panel.

Weanling, WT and *Hamp*-KO mice were fed diets with variable NHI content (5–50 ppm) for 6 weeks (Figure 5A). Consumption of the diets with 30 and 50 ppm total iron caused iron loading in *Hamp* KOs, as indicated by elevated serum ferritin, a biomarker for body iron stores (49), and increased liver iron content, as compared with WT mice consuming the same diets. *Hamp*-KO mice consuming the 5- and 15-ppm NHI diets were, however, protected from iron overload (Figure 5, B–F). Given these outcomes, a subsequent iron loading study was undertaken using NHI (control) and HI-enriched (experimental) diets with 50 ppm total iron. The use of an internal control diet with 7 ppm NHI, to match the NHI content of the HI-enriched diet, was deemed unnecessary. Outcomes from a subsequent 6-week, iron-loading study showed that *Hamp*-KO mice consuming both diets had elevated TSAT, and higher serum and tissue NHI levels, indicative of iron overload (Figure 6). In the liver, pancreas, and heart, iron loading was more pronounced in the NHI diet group, and iron loading in the kidney was only seen in this group. Nonetheless, these data demonstrated that NHI and HI both contribute to iron loading in *Hamp*-KO mice, consistent with observations made in humans (45, 46). These data further supported the concept that, under these experimental conditions, B6 mice assimilated iron from dietary heme.

**Figure 7:**
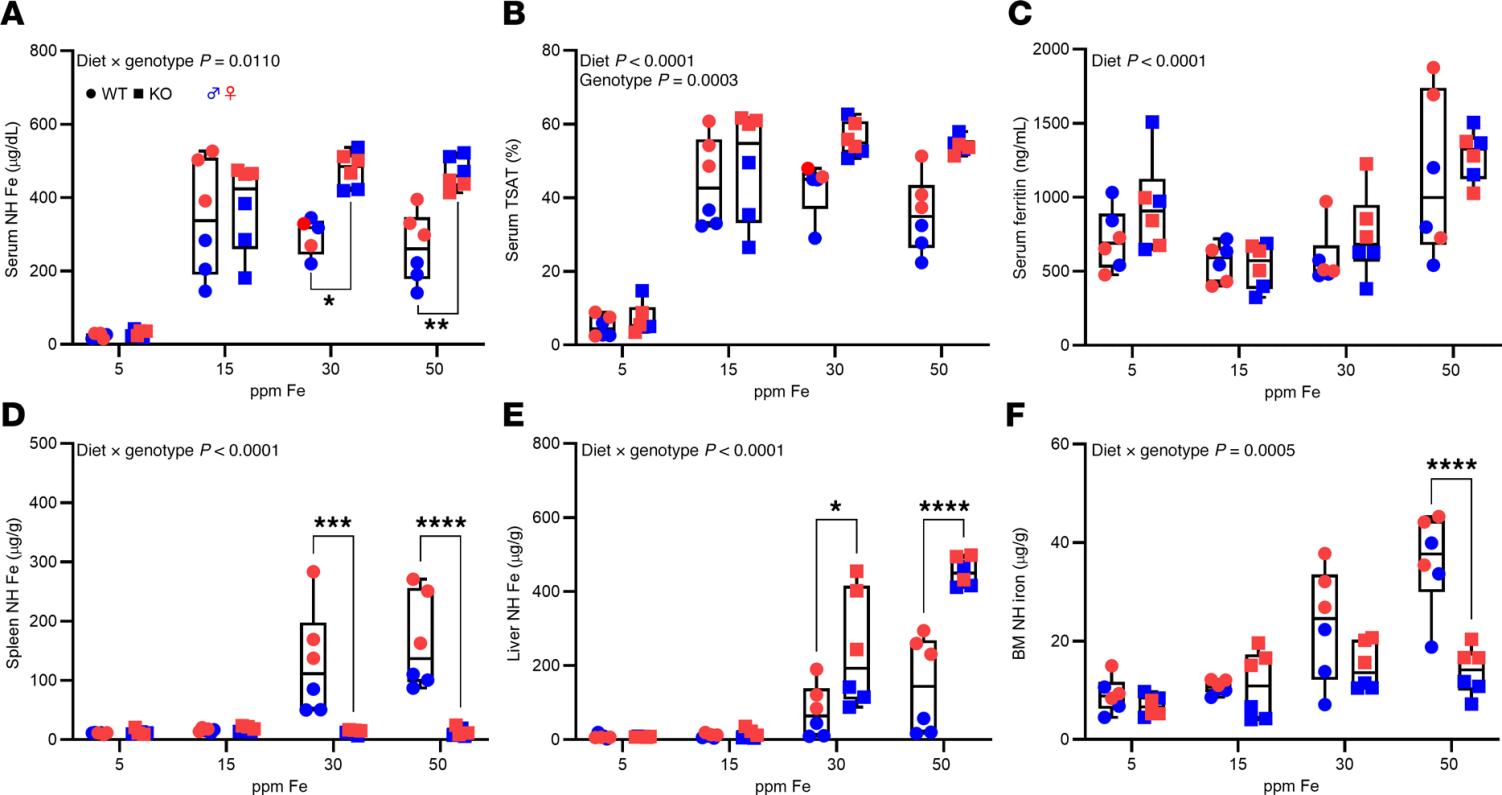
Consumption of a 15-ppm NHI diet prevents iron overload in *Hamp*-KO S-D rats. WT and KO rats of both sexes were fed diets with variable NHI content for 6 weeks after weaning and then sacrificed for analysis. Shown are serum NHI levels (**A**), TSAT (**B**), serum ferritin (**C**), and spleen (**D**), liver (**E**) and bone marrow (**F**) NHI content. Data are presented as box-and-whisker plots for *n* = 5–6 rats/group and were analyzed by 2-way ANOVA followed by Tukey’s multiple-comparison test. Asterisks denote significant differences between the WT and KOs within a diet group. *P < 0.05; **P < 0.01; ***P < 0.001; ****P < 0.0001. Significant 2-way interaction and main effect *P* values are shown in each panel.

**Figure 8:**
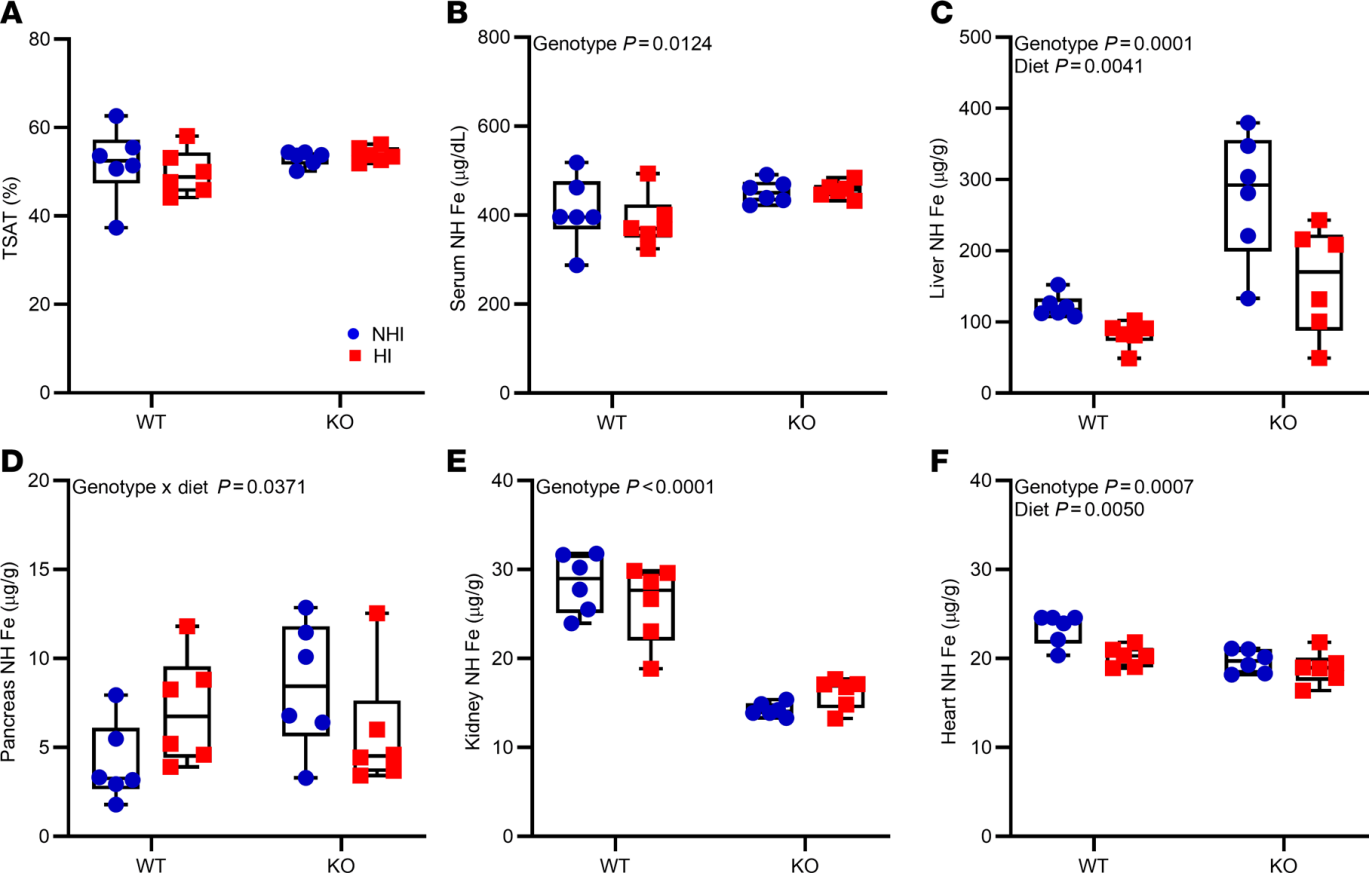
Consumption of a 50-ppm NHI diet or a 50-ppm HI–enriched diet leads to iron loading in *Hamp*-KO S-D rats. Female WT and *Hamp*-KO rats were weaned onto a control diet with 50 ppm NHI (blue circles), or an experimental 50-ppm HI–enriched diet (red squares), and then sacrificed after 6 weeks on the diets. Shown are serum TSAT (**A**), and serum (**B**), liver (**C**), pancreas (**D**), kidney (**E**), and heart (**F**) NHI levels, which were determined in experimental rats at the termination of the experiment. Results are presented as box-and-whisker plots for *n* = 6 rats per group. Data were analyzed by 2-way ANOVA followed by Tukey’s multiple-comparison test. Groups with different letters vary significantly. Significant 2-way interaction and main effect *P* values are shown in each panel.

Parallel experiments in *Hamp*-KO S-D rats showed similar outcomes. As in mice, consumption of the 50- and 30-ppm NHI diets led to increases in serum and liver iron content, and TSAT, indicative of iron loading in the KOs, but rats consuming the 5- and 15-ppm NHI diets did not load excess iron (Figure 7). As in mice, iron overload was more pronounced when rats consumed the 50-ppm iron diet. A subsequent iron loading study showed that both the NHI and HI-enriched diets with 50 ppm total iron increased serum and liver iron levels in *Hamp* KOs (Figure 8). Changes in pancreatic and heart iron levels, although statistically significant, were minor and less likely to be biologically relevant. Renal iron content was lower in both KO groups, but no differences were noted when comparing diets within each genotype. NHI and HI thus both contributed to the iron loading caused by excessive intestinal iron absorption in *Hamp*-KO S-D rats and B6 mice.

## Discussion

The ability of humans to utilize iron derived from dietary heme was first described in 1955 (50). Many additional studies on HI utilization, performed in various model organisms including dogs, rats, and pigs, and in humans, were published in subsequent decades; however, despite over 60 years of intensive research, specific mechanisms of HI absorption remain to be defined (14). In recent years, most studies on iron biology have shifted to mice (27), even though mice (31) are thought to inefficiently assimilate iron from dietary heme (28, 29). This generalized perception has impeded the development of new rodent models of HI absorption. Our goal in this investigation was to develop a nutritional approach to test HI absorption in rats and mice under conditions in which iron absorption is elevated, including IDA, pregnancy, and HH. We hypothesized that providing iron as Hb in the context of an ad libitum–fed, AIN-93G–based rodent diet would be the best approach to test HI absorption and utilization in rodents. Several recently published reports demonstrated HI utilization by rats (30, 31, 33, 35, 36), but with variable efficiencies being reported, so the first objective was to identify a rat strain that could more effectively utilize dietary Hb as an iron source. Initial testing in 3 rat strains rationalized our choice of S-D rats for further experimentation.

The experimental approach utilized here was to formulate rodent diets containing either HI (as porcine RBCs) or NHI (as ferrous sulfate). Lyophilized porcine RBCs provide iron mainly as Hb. We reasoned that providing HI as Hb would best mimic natural dietary sources and promote absorption, since peptides produced from the proteolysis of Hb have been shown to increase solubility and enhance absorption of dietary heme (35, 51). Diets with NHI content matching the background NHI content in the HI-enriched diets were utilized as internal controls. The total iron content of the diets encompassed the normal range of required iron intake for rodents (40, 41). Using diets containing both main forms of iron mimics an omnivorous human diet, thus increasing translational relevance. These experimental diets were utilized to test whether HI absorption was sufficient to (a) allow recovery from IDA; (b) provide the extra iron required to support pregnancy, lactation, and neonatal development; and (c) precipitate iron loading in *Hamp* KOs.

Iron depletion-repletion experiments revealed that a 75-ppm HI–enriched diet was just as effective as a 50-ppm NHI diet at correcting the iron deficiency and anemia in S-D rats. Since the background NHI level in the 75-ppm HI–enriched diet was only approximately 11 ppm and the estimated dietary iron requirement for a growing rat is 35 ppm (40), this outcome provided strong evidence that dietary HI was efficiently absorbed and utilized by S-D rats. Moreover, NHI and HI-enriched diets with 50 ppm total iron adequately supported many aspects of normal pregnancy. Dams in the HI group, however, weighed less (by ~20%) and had lower serum iron (~60% lower) than controls, despite having elevated iron stores in the spleen (2-fold higher) and liver (2.9-fold higher). Hypoferremia in dams could have been the cause of the noted slight growth impairment and (nonsignificant) trends toward decreases in Hb, Hct, serum iron, and TSAT in their pups. Enhanced iron uptake by the liver and spleen in dams consuming the HI diet could be indicative of the extramedullary hematopoiesis that typifies pregnancy (52). The fact that this phenomenon was only observed in the HI diet group may indicate that dietary iron was below the absolute requirement. Supporting this possibility, 75 ppm total iron was required in an HI-enriched diet to allow recovery from IDA in S-D rats. Furthermore, iron loading studies revealed that serum iron was similarly elevated in *Hamp*-KO rats in both dietary groups, as was hepatic iron. Collectively, these experiments provide strong evidence that S-D rats can assimilate and utilize dietary iron derived from heme.

Given these notable experimental outcomes in rats, subsequent experiments in mice were warranted. In mice, a 60-ppm HI–enriched diet was as equally effective as the 50-ppm NHI control diet at correcting the anemia in iron-depleted mice. All other iron and hematological indices were fully restored in both dietary groups, except for liver iron stores, which remained significantly lower in the HI diet–fed mice. However, since serum iron, splenic iron, and liver *Hamp*, renal *Epo*, and bone marrow *Erfe* expression levels were fully restored to control values, we conclude that HI can be sufficiently absorbed in B6 mice to correct severe IDA. Moreover, in pregnant mice, the 50-ppm HI and NHI diets were equally effective at supporting fetal and neonatal outcomes. Additional experimentation established that mice accumulated excess iron in serum, liver, pancreas, kidney, and heart when fed HI or NHI diets with 50 ppm total iron content. Tissue iron loading was more significant when mice consumed the NHI diet, suggesting that this form of iron may be better absorbed in this model of HH under these experimental conditions.

In summary, dietary HI was effective at correcting IDA and supporting pregnancy, lactation, and pup development in WT S-D rats and B6 mice, and HI also contributed substantially to the iron overload that typifies murine HH. These outcomes, however, contrast with previous studies suggesting inefficient HI absorption by rats (9, 13, 29, 53, 54) and mice (28, 29, 55, 56). Different results may have been obtained in the current study since the experimental approach, which included HI as mainly Hb within porcine RBCs in the context of semipurified, defined rodent diets, was unique among previously published papers on this topic. Identifying appropriate rat and mouse strains and defining a nutritional/physiological paradigm in which to study HI absorption should provide new opportunities to experimentally address fundamental uncertainties in this important area of research.

## Methods

### Sex as a biological variable.

In most experiments, female rats and mice were used, while in one experiment, both sexes were studied. We did not have a preconceived, scientific rationale for using females, but we have noticed that many iron biology studies over the past 2 decades have used female mice (for perhaps unexplained reasons). Possible outcomes in male rats and mice are difficult to predict but would probably be quite similar (based upon our interpretation of the primary, scientific literature, and our own personal experiences).

### Experimental animals and diets.

Rats and mice utilized for these studies were purchased from Charles River Laboratories or bred at the University of Florida Animal Care facilities. Semipurified, AIN-93G–based diets, containing either NHI as FeSO_4_ or HI derived from lyophilized porcine RBCs (a kind gift from APC, Inc.), were formulated and manufactured by Dyets, Inc. The lyophilized porcine RBCs contained approximately 85% HI and 15% NHI, as determined by ICP/MS and UPLC analysis (Center for Iron and Heme Disorders, University of Utah). The iron content of all other dietary components, including casein, iron-free mineral mix, FeSO_4_ mix, and the basal diet, was determined by Eurofins, to ensure accuracy of the final, manufactured diets. To prevent coprophagia (and mineral recycling), rats and mice were housed in overhanging wire-bottom, stainless-steel cages during iron depletion periods (when animals consumed a low-iron diet with ~3 ppm NHI).

### Rat pilot studies.

In rat pilot study 1, a preliminary depletion/repletion trial was conducted in 3 common laboratory rat strains, S-D, Lewis, and Wistar. Weanling, female rats of each strain were randomized into groups and fed a low-iron diet (3 ppm NHI) for 2 weeks to induce IDA. Subsequently, they were repleted with either an iron-adequate 50-ppm NHI (FeSO_4_) diet, an HI-enriched diet (porcine RBCs; 43 ppm HI + 7 ppm NHI), or an internal control diet with 7 ppm NHI (to match the background NHI content in the HI-enriched diet). Additional control groups were maintained on either an adequate-iron diet with 50 ppm iron or a low-iron diet with 3 ppm iron throughout the study. Blood was drawn by venosection to assess Hb levels just before (0 hours) and by cardiac puncture at the termination of the experiment (84 hours). Results of this experiment showed that among the 3 tested strains, S-D rats had the greatest increase in Hb after consuming the HI repletion diet, so S-D rats were selected for further experimentation.

In rat pilot study 2, a more extensive iron depletion/repletion study was undertaken in S-D rats. The study design and diets used are exactly as described above, but the repletion period was extended out to 26 days. Blood samples were collected from the tail vein at baseline and every 4 days thereafter to monitor blood Hb levels. At the end of the study, blood was collected from CO_2_-anesthetized rats via cardiac puncture, just prior to euthanasia by thoracotomy. Subsequently, various organs and blood were removed and stored at –80°C. Biochemical assessments included Hb concentration in whole blood, serum, liver, and spleen NHI content, and TSAT (as detailed below).

### S-D rat depletion-repletion study using experimental diets with adjusted iron content.

Our initial studies showed that a 50-ppm HI diet fully corrected the anemia in iron-depleted S-D rats, but liver iron was not fully restored, indicating that rats remained iron deficient. We thus designed a subsequent study using an identical experimental approach but adjusting the HI diets up to 100 ppm total Fe (see experimental design in Supplemental Table 1). The same iron-adequate and iron-deficient control groups were included, and also a 15-ppm NHI repletion diet that served as an internal control for the 100-ppm HI diet (which contained ~15 ppm NHI). Iron-depleted, anemic female S-D rats were repleted with the different diets for 8 days (until the anemia was completely resolved in some groups). Outcome assays included Hb and Hct from whole blood, serum EPO and hepcidin concentration by ELISA, serum and tissue NHI content, TSAT, and liver *Hamp*, renal *Epo*, and bone marrow *Erfe* mRNA levels.

### Mouse iron depletion-repletion study.

The physiological/nutritional approach utilized here successfully revealed efficient HI utilization by S-D rats, leading us to predict that similar outcomes would also be seen in mice, since both species share a common evolutionary history as opportunistic omnivores. We (logically) chose the C57BL/6 strain, which is commonly used for iron-related (patho)physiology studies. The experimental approach was identical to that used for rat studies (see experimental design in Supplemental Table 2), but the low-iron feeding period was extended to 3 weeks since it takes longer to deplete B6 mice of iron by dietary means (our personal observations). Blood was withdrawn by venipuncture every 4 days during the depletion period to track changes in blood Hb levels. Mouse pilot study 1 utilized the same diets described above for the rat depletion/repletion experiments, with experimental HI-enriched diets ranging from 50 to 100 ppm total iron. Outcomes, however, showed that all experimental diets very rapidly resolved the anemia in mice, indicating that the dietary iron concentrations were too high. Mouse pilot study 2 was thus undertaken with iron from 15 to 30 ppm; however, total dietary iron up to 30 ppm was insufficient at correcting the anemia in iron-depleted mice. So finally, new HI-enriched diets were formulated with total iron up to 60 ppm. An internal control diet contained 9 ppm NHI as ferrous sulfate (to match the NHI content in the 60-ppm HI-enriched diet). Outcomes assays were the same as those described above.

### HI pregnancy study in rats and mice.

The purpose of this study was to examine whether a HI-based diet could provide the additional iron required to support pregnancy, lactation, and pup development. We postulated that a physiological/nutritional approach using an HI-enriched, AIN-93G–based diet would support normal pregnancy outcomes in S-D rats and B6 mice. Accordingly, we compared the effects of iron-adequate, 50-ppm HI-enriched (experimental) and NHI (control) diets on pregnancy. Twenty-four, 8-week-old S-D rats (12 females and 12 males) were purchased from Charles River Laboratories for the study. Rats were fed experimental diets for 7 days prior to and during a 14-day mating period, after which the males were removed. Dams were kept on their respective diets throughout gestation and lactation. Body weights and Hb levels were not measured during the fetal period to prevent subjecting the rats to undue stress. At the end of the weaning period (after ~21 days of lactation), dams and pups were sacrificed for further analysis (4 pups; 2 males and 2 females per dam, randomly selected). The litter size, sex distribution of pups at birth, and final body weights of dams and pups were measured. The following iron-related parameters were assessed using standard experimental protocols: Hb and Hct from whole blood, serum NHI content, and TSAT. NHI content in the liver and spleen was also determined. The study was repeated in B6 mice, using the exact same experimental design and approach.

### Hamp-KO mouse and rat iron loading study.

Our first objective was to determine the minimum level of dietary iron that causes iron overload in *Hamp*-KO rats and mice (since these animals hyperabsorb dietary iron). This experiment was important to inform the rationale design of subsequent iron loading experiments. *Hamp*-KO, C57BL/6 mice were a gift from Sophie Vaulont at the Institut Cochin, Paris, France (47), while *Hamp*-KO, S-D rats (48) are maintained by us in house. WT and *Hamp*-KO mice of both sexes were weaned onto 1 of 4 AIN-93G–based, semipurified rodent NHI diets, with the following target iron concentrations: 5 (low), 15 (low marginal), 30 (high marginal), or 50 (adequate) ppm. Weanling animals were fed these different diets for 6 weeks. Standard experimental protocols were used to assess the following iron-related parameters: serum NHI content, TSAT, and serum ferritin concentration. Iron content in spleen and liver was also determined. This study was repeated using both WT and *Hamp*-KO rats of both sexes. The same iron-related parameters were measured, with the addition of bone marrow NHI levels in the rat study.

The next experimental goal was to test whether dietary HI causes iron loading in *Hamp*-KO mice. A total of 24 female, 3-week-old WT and *Hamp*-KO mice were weaned onto either a 50-ppm HI–enriched diet or a 50-ppm NHI diet for 6 weeks. Subsequently, mice were euthanized for further analysis. Standard experimental protocols were used to assess serum NHI content and TSAT. NHI content in the liver, pancreas, kidney, and heart was also quantified. This exact same study was also conducted in WT and *Hamp*-KO S-D rats, to evaluate whether HI and NHI both contribute to iron loading in this HH model.

### Hematological and iron-related parameters.

A standard method was used to measure Hb and Hct in whole blood (57). Blood was transferred to a serum separation tube (365967, BD Diagnostics), which was centrifuged at 12,000*g* for 2 minutes to separate the serum. Serum NHI levels were determined using a colorimetric method (58). Total iron-binding capacity (TIBC) was measured using a previously described colorimetric method (59, 60). Percentage TSAT was calculated as serum iron/TIBC × 100. To measure NHI concentrations in tissues, a standard colorimetric method was used, as described in a previous study (61). Briefly, a snap-frozen tissue sample (~40 mg) was treated with an acid solution containing 3 M hydrochloric acid and 10% (w/v) trichloroacetic acid at 65°C for 20 hours. A portion of the resulting supernatant was combined with a working chromogen reagent composed of 0.1% (w/v) bathophenanthroline sulfonate (BPS), 1% (v/v) thioglycolic acid in iron-free water, and saturated sodium acetate at a ratio of 1:5:5. The absorbance (OD) of the mixture was measured at 535 nm using a Synergy H1 plate reader (BioTek) and compared to a certified iron reference solution (Thermo Fisher Scientific).

### ELISA.

The levels of serum EPO (MEP00B, R&D Systems), serum hepcidin (HMC-001, Intrinsic Lifesciences), and serum ferritin (ab157713, Abcam) were assessed using ELISA following instructions provided by the manufacturer.

### Quantitative real-time PCR.

RNA was extracted from tissues using RNAzol RT reagent (Molecular Research Center, Inc.) as per the manufacturer’s instructions. The RNA concentration was measured using a Nanodrop spectrophotometer, and RNA quality was evaluated by agarose gel electrophoresis. SYBR-Green qRT-PCR was performed using a well-established protocol (62, 63). Fold changes of mRNA expression for each specific gene were calculated using the 2^−ΔΔCt^ analysis method. The expression level of target genes was normalized to those of the reference gene cyclophilin A (*CypA*). Primer sequences are listed in Supplemental Table 3.

### Statistics.

Results are presented as line plots illustrating mean ± SD, or box-and-whisker plots illustrating the median (line in box), interquartile range (IQR, bounds of the boxes), and min and max values (whiskers). Statistical analysis was carried out using GraphPad Prism (version 8.4). Data were analyzed by 1- or 2-way ANOVA. If significant main effects or interactions were observed, Tukey’s multiple-comparison post hoc test was performed to compare individual groups. A *P* value of less than 0.05 was considered statistically significant.

### Study approval.

All experimental procedures involving animals were approved by the University of Florida IACUC.

### Data availability.

Values for all data points in graphs are reported in the Supporting Data Values file.

## Author contributions

JKL, SRLF, IH, and JFC conceptualized and designed the study. JKL, SRLF, YH, RRW, XW, JSS, SZ, POEU, SS, MF, and YY performed experiments, analyzed data, and designed subsequent experimental approaches. JKL, SRLF, IH, and JFC designed figures and wrote the draft of the manuscript. All authors approved the final version of the submitted manuscript.

## Supplementary Material

Supplemental data

Supporting data values

## Figures and Tables

**Table 4:**
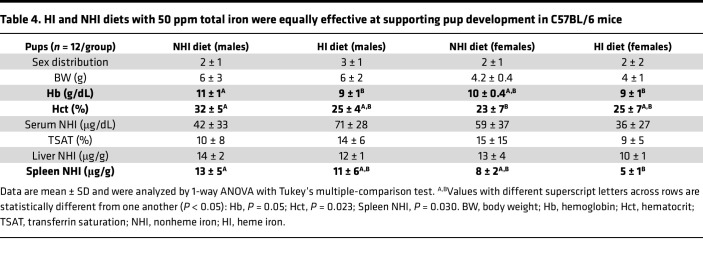
HI and NHI diets with 50 ppm total iron were equally effective at supporting pup development in C57BL/6 mice

**Table 3:**
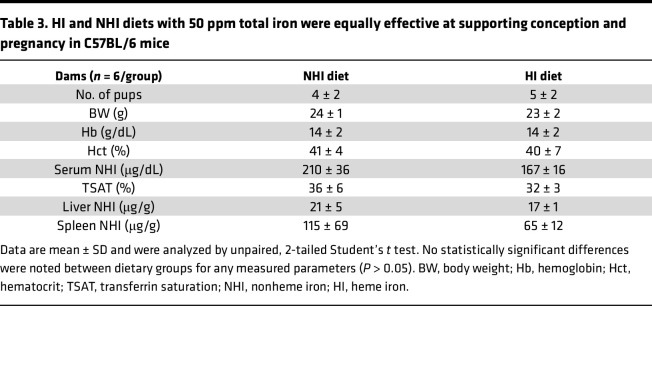
HI and NHI diets with 50 ppm total iron were equally effective at supporting conception and pregnancy in C57BL/6 mice

**Table 2:**
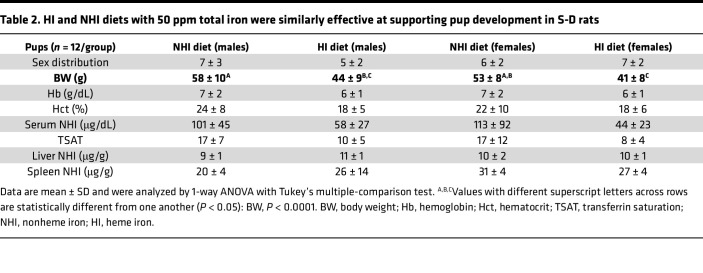
HI and NHI diets with 50 ppm total iron were similarly effective at supporting pup development in S-D rats

**Table 1:**
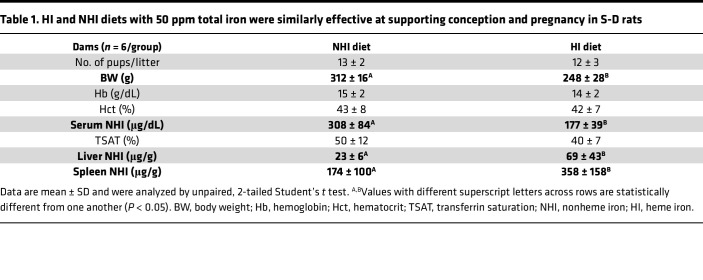
HI and NHI diets with 50 ppm total iron were similarly effective at supporting conception and pregnancy in S-D rats

## Version 1 06/09/2025

Electronic publication
