# Supplementary material for: Development of rat and mouse models of heme-iron absorption
Source: JCI Insight. 2025 Jun 9;10(11):e184742. doi: 10.1172/jci.insight.184742 (PMC12220949; doi:10.1172/jci.insight.184742)
Supplement: Supplemental data [file jciinsight-10-184742-s134.pdf]

Supplemental Materials

Lee et., al. Development of Rat and Mouse Models of Heme-iron Absorption. *JCI Insight*

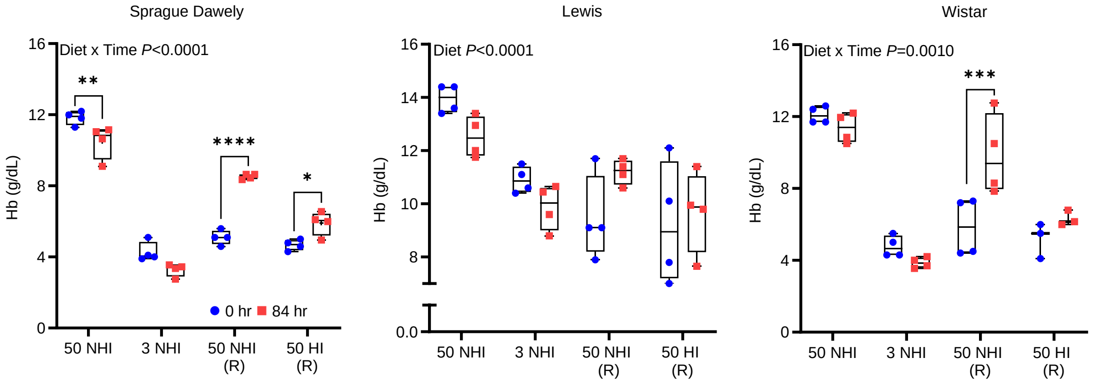

Figure S1. Rat Pilot Study One: Sprague-Dawley rats utilized dietary HI more efficiently than Lewis or Wistar rats. Weanling, female rats were fed a low-iron diet (3 ppm NHI) for two weeks to induce anemia, and then repleted (R) with 50 ppm HI-enriched or NHI diets for 84 hours. Control groups were fed an adequate-iron diet (50 ppm HI) or the low-iron diet throughout. Hemoglobin (Hb) levels were quantified in rats at the termination of the experiment. '0 hr' indicates animals that were killed prior to the repletion period, while '84 hr' indicates rats killed at the end of the repletion period. Data are mean ± SD for n=4 rats/group and were analyzed by two-way ANOVA followed by Sidak's multiple comparisons test (*$p<0.05$; **$p<0.01$; ****$p<0.0001$). Significant two-way interaction and main effect *P* values are shown in each panel.

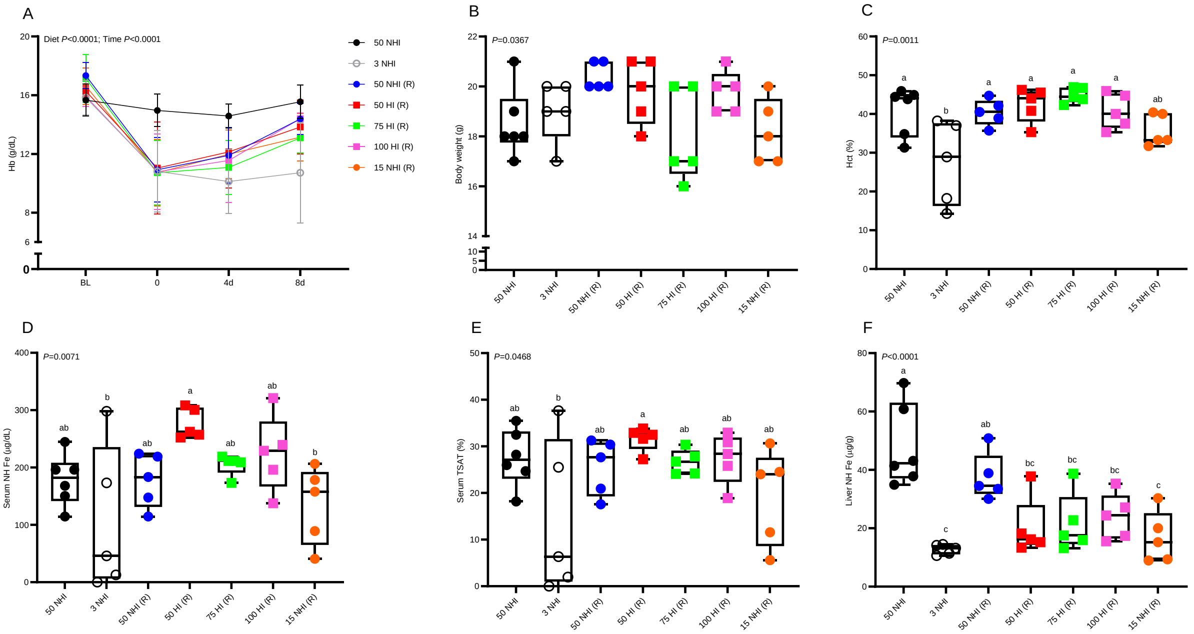

Figure S2. Mouse Pilot Study One: Experimental HI diets and the control NHI diet were equally effective at correcting the anemia in iron-depleted C57BL/6 mice. Weanling, female mice were fed a low-iron diet (3 NHI) for 3 weeks and then repleted with 50, 75 or 100 ppm HI experimental diets, or a 50 ppm NHI (iron adequate) diet. The 15 ppm NHI diet matched the NHI content of the 100 ppm HI diet (85% HI and 15% NHI). Control groups were fed the iron-adequate or the low-iron diet throughout. Mice were bled every 4 days and killed 8 days after initiation of the iron-repletion phase. Hemoglobin levels are shown at each time point (**A**). Also shown are body weight (**B**), hematocrit (**C**), serum NHI levels (**D**), serum TSAT (**E**), and liver (**F**) and spleen (**G**) NHI levels in experimental mice at the termination of the experiment. Results are presented as a line graph (A) or boxplots for n=5-6 mice per group. Data were analyzed by one- (B-G) or two-way (A) ANOVA followed by Tukey's multiple comparisons test. Groups with different letters vary significantly. Significant two-way interaction (A) and main effect (B-G) *P* values are shown in each panel. Panel A: BL, baseline, prior to iron deprivation; 0, after three weeks of low-iron feeding; 4d, 8d indicates repletion (R) period in days.

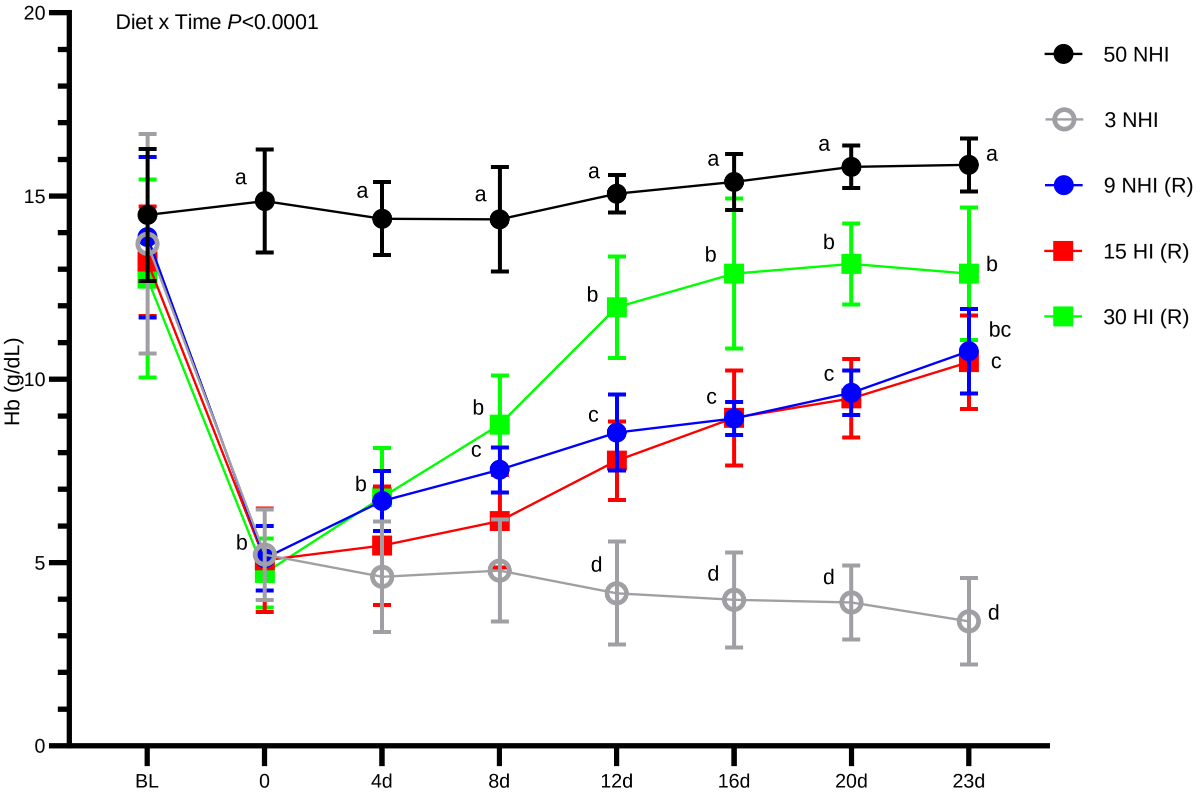

Figure S3. Mouse Pilot Study Two: A 30 ppm HI diet was ineffective at correcting the IDA in iron-deprived C57BL/6 mice. Weanling, female mice were fed a low-iron diet (3 ppm NHI) for 3 weeks and then switched to repletion diets, including 9 ppm NHI (internal control), and 15 and 30 ppm HI (experimental). Control groups consumed an iron-adequate diet (50 NHI) or the low-iron diet throughout. Mice were bled every 4 days and killed 23 days after initiation of the iron-repletion phase. Hemoglobin levels are shown at each time point. Data were analyzed by two-way ANOVA followed by Tukey's multiple comparisons test (n=6 mice per group). Groups with different letters vary significantly at each time point. A significant two-way interaction *P* value is shown in the panel. BL, baseline, prior to iron deprivation; 0, after three weeks of low-iron feeding; 4d, 8d, etc., indicates repletion (R) period in days.

Table S1. Experimental design for HI absorption and utilization studies in SD rats.

Age (days)	Replete Control	Depleted Control	Control Repletion	Experimental Repletion Groups			
21-35	50[1] NHI (Ad Fe)	DEPLETION 3 NHI (Low Fe)	DEPLETION 3 NHI (Low Fe)	DEPLETION 3 NHI (Low Fe)			
36-43			REPLETION 50 NHI	REPLETION 50 HI	REPLETION 75 HI	REPLETION 100 HI	REPLETION 15.1 NHI

[1]Target concentrations of iron (in ppm). **NHI**, non-heme iron ($FeSO_4$); **HI**, heme iron (porcine RBCs); **Ad Fe**, adequate iron.

Table S2. Experimental design for HI absorption and utilization studies in B6 mice.

Age (days)	Replete Control	Depleted Control	Control Repletion	Experimental Repletion Groups				
21-42	50[1] NHI (Ad Fe)	DEPLETION 3 NHI (Low Fe)	DEPLETION 3 NHI (Low Fe)	DEPLETION 3 NHI (Low Fe)				
43-66			REPLETION 50 NHI	REPLETION 15 HI	REPLETION 30 HI	REPLETION 60 HI	REPLETION 9 NHI	

[1]Target concentrations of iron (in ppm). **NHI**, non-heme iron ($FeSO_4$); **HI**, heme iron (porcine RBCs); **Ad Fe**, adequate iron.

Table S3. Oligonucleotide primers used for qRT-PCR analyses

Gene	Direction	Sequence (5' to 3')
Hamp	Forward	GGCAGAAAGCAAGACTGATGAC
Hamp	Reverse	ACAGGAATAAATAATGGGGCG
Epo	Forward	AGTCGCGTTCTGGAGAGGTA
Epo	Reverse	ACTTTGGTATCTGGGACGGTAA
Erfe	Forward	ACTCACCAAGCAGCCAAGAA
Erfe	Reverse	TTCTCCAGCCCCATCACAGT
Cyclophilin	Forward	CTTGCTGCAATGGTCAACC
Cyclophilin	Reverse	TGCTGTCTTTGGAACTTTGTCTGC
